# Governing policies and factors affecting the labor market integration of female accompanying spouses

**DOI:** 10.3389/fsoc.2022.1084390

**Published:** 2023-01-12

**Authors:** Musawenkosi Donia Saurombe, Farirai Zinatsa

**Affiliations:** ^1^Department of Industrial Psychology and People Management, College of Business and Economics, University of Johannesburg, Johannesburg, South Africa; ^2^Centre for Development Support, Faculty of Economic and Management Sciences, University of the Free State, Bloemfontein, South Africa

**Keywords:** labor market integration, skilled migration, accompanying spouse, female, governmentality, conduct of conduct

## Abstract

**Introduction:**

This study sought to ascertain the macro governing policies and factors that influence the integration of female accompanying spouses in the Free State, into the South African labor market.

**Methods:**

Utilizing a qualitative approach, thirteen one-on-one interviews, consisting of an initial purposive sample and a subsequent snowball sample, were conducted for data gathering. The study employed thematic analysis to interpret the data.

**Results:**

The findings revealed that governing policies emerging from South Africa's migration legislation, and factors such as spouse dependence, reinforcement of traditional gender roles, and restrictive employment legislation which forced deskilling of qualifications, mainly impacted the conduct of accompanying spouses concerning the labor market.

**Discussion:**

This study contributes to the literature on labor market integration (LMI) from an underexplored South-to-South standpoint by delving into the experiences of skilled female migrants in the family migration setting. A neglected facet of Michel Foucault's governmentality theory was used to investigate the labor market assimilation needs of female accompanying spouses. The study's qualitative approach renders the findings much less generalizable than a quantitative inquiry. It is important to note that LMI research is considerably setting-specific, despite some aspects of this study being applicable to other settings in the Global South. South Africa continues to be a pivotal regional hub for migration in the Global South, yet it has a complex migration governance framework that sets up a specific, while broadly exclusionary, macro context for accompanying spouses. This study zones in on issues that could inform more effective family migration policy.

## 1. Introduction

Labor migration has pervaded South Africa's economy for several decades. A fairly steady economy, political stability and a considerable respect for human rights are some of the key aspects contributing promoting post-apartheid South Africa as a pivotal regional migration hub and a favored destination for numerous labor migrants. Notwithstanding, the migration governance framework in South Africa has been criticized for being contentious and contradictory, as substantiated by related policies that are both inclusive and constricting (Amit and Kriga, 2014; Van Lennep, 2019). Although the positive role of migrants within the South African economy is evident [The Organization for Economic Co-operation and Development-International Labor Organization (OECD/ILO), 2018a, Vermaak and Muller, 2019], widespread misconceptions prevail about migrants “stealing” the jobs of South Africans, placing unnecessary strain on public services, and participating in unlawful activities (Adjai and Lazaridis, 2013; Amit and Kriga, 2014; Kanayo et al., 2019). For several years now, South Africa has leveraged its migration policies to deal with challenges regarding labor shortages and to manage the available labor pool. Post-2000, the country adopted a skills-based immigration policy to facilitate skilled migration while increasing semi-skilled or unskilled restrictions. However, policies centered on economic development are broadly criticized for failing to account for the complexities associated with family migration (Holliday et al., 2019).

Women who migrate within the context of family as accompanying spouses often suffer the migration consequences to the greatest extent (Ballarino and Panichella, 2017). Many of them can be regarded as economic or skilled migrants, with aspirations to be integrated into the labor market (Riaño, 2016). Consequently, accompanying spouses seeking employment are subjected to navigating a complex array of policies, regulations, practices, and narratives of labor migration, which inevitably frames, shapes, and controls their behavior regarding the labor market in South Africa. Additionally, accompanying spouses continue to be fundamentally invisible in migration studies and the prevailing gender inequalities in migration research are mostly to the disadvantage of women. For instance, Brieger and Gielnik (2021) found that female migrants are less likely to start or run their own businesses than their male counterparts, and female accompanying spouses often prioritized entrepreneurship in host countries due to their limited labor market integration (LMI) prospects compared to male migrants. ILO (2016) and the Gereke et al. (2020) further reveal how female migrants constitute about half of international migration worldwide, yet the character of female migration has not changed much since the beginning of the era of mass global migration because data systems are gender-blind, mostly in favor of men. The ILO (2016) also suggest how women largely work in “invisible” sectors and do not enjoy the same labor legislative protection as their male counterparts do. It is therefore imperative for research to be undertaken to unearth various LMI inhibiting factors which could possibly be considered when drafting and amending labor migration policy in the host country.

The theory of governmentality by Michel Foucault was adopted in this research. In *Security, Territory and Population*, Foucault (1978) coined governmentality as the conduct of conduct. Governing involves the use of vast techniques, practices, narratives, or strategies to model and adjust people's behavior toward specific outcomes (Lemke, 2002; Korteweg, 2017). Such techniques are crafted through mentalities or thoughts (governmentalities) which presuppose that conduct is deliberately shaped rather than randomly or arbitrarily. Rajas (2012) posits that governmentality proffers the notion that some ways of being are better to others. Thus, governmentality functions through the shaping of the subjectivities of those being governed. Certain personal subjectivities are because of specific knowledges that determine possibilities (Ho, 2017). For instance, in South Africa, the legal label of “dependent” assigned to accompanying spouses influences their ability to work legally. Consequently, governmental rationalities can be broadened into individual lives and society at large (Rajas, 2012). From a Foucauldian standpoint, power is re-crafted as power *from afar* that entails the shaping of behaviors, aspirations, values, and education preferences. In relation to this, the exercise of power is observable through the voluntary behavior of the one being governed (Del Percio, 2018).

Migration in the Global South remains under-researched (Bastia and Piper, 2019; Gisselquist and Tarp, 2019) and there are proven gaps in the literature particularly concerning migration by skilled female labor. Most research investigating and exploring the experiences of immigrant women has mostly placed emphasis on women who are socio-economically disadvantaged, specifically, refugees or those seeking asylum status, as well as unskilled and semi-skilled migrants, as a result, there is a dearth of research on skilled women migrants (Kaushik and Walsh, 2018). The purpose of this research was to ascertain the macro governing policies and factors influencing the integration of accompanying spouses by deconstructing the experiences of specifically skilled female accompanying spouses concerning LMI in South Africa. The study specifically considered the perspectives of accompanying spouses in the Free State province.

## 2. A brief literature review

### 2.1. Governmentality and migration policy in South Africa

Michel Foucault is considered as one of the leading scholars who highlighted the techniques and strategies of power, specifically, how power is exercised. Regarding governmentality, Foucault denotes structured attitudes, applications and rationalities through which individuals, or structures are governed (Foucault, 1978). Governmentality is framed as the “conduct of conduct” [(Foucault, 1978), p. 220–1], which oscillates between “governing self to governing others.” It denotes any effort to craft behavior in line with a particular assortment of norms and for various outcomes through considerable forethought and consideration (Lemke, 2012).

Immigration centers on biopolitical rationalities that are primarily associated with the management of the cross-border movements of migrants. In this regard, South Africa places a strong emphasis on the notion of “sovereignty” which is regarded as the right to determine who and who is not eligible to pass through its borders (Department of Home Affairs, 2017). In line with economic development goals, there has been a concerted effort to enable the mobility of exceptionally skilled and qualified people into South Africa. Rajas (2012) argues that it is not sufficient to consider biopolitical rationalities alone: those that concern the management of the integration of migrants into society must also be considered. Due to the inextricable and enduring bond between labor and migration policies in South Africa, one could argue that the rationalities that impact cross border mobility equally affect the LMI of migrants (Ala-mantila and Fleischmann, 2018). Governmentalities find expression in aspects such as policy frameworks that directly impact migrant outcomes as they determine what opportunities may be available for migrants to capitalize on in the host country (Bhattacharjee, 2017).

Beginning in the colonial era right through to that of post-independence, the regulation of cross border control in South Africa was fueled by segregationist and exclusionary rationalities. Racially discriminatory practices characterized labor migration (Statistics South Africa, 2018), which ensured continued domination by colonizers and an abundant quota of cheap labor migrants (Department of Home Affairs, 2016). Migrants, particularly those of African descent, continue to be subjected to different forms of prejudice, discrimination and even violence. Korteweg (2017) note that even institutions (including workplaces) became sites of exclusion under xenophobia. With greater acceptance of the role that skilled migration could play in development, South Africa began to open the borders to Africans and the world but with the important caveat of reserving the right of entry mainly to highly skilled immigrants or those with critical skills (Department of Home Affairs, 2016). Those from the South African Development Community (SADC), regarded as having low to mid-level skills, could only work on farms, mines, and other companies under a temporary Corporate Work Visa (Department of Home Affairs, 2016).

The Draft Green Paper for International Migration signified a significant shift in policy rationale. A memorandum of understanding between the government of South Africa and the Southern African Development Community and the UN High Commission for Refugees culminated in a more open, rights-based approach to migrants and refugees (Amit and Kriga, 2014). The 1998 Refugees Act and the subsequent 2002 Immigration Act heralded a new era of migration policy. Though strong views continued around undesirable, unauthorized immigration, immigration was cast in a more favorable light as having the potential to be a tool for nation-building instead of being an impediment.

Eradicating xenophobia was an explicit goal of the 2002 Immigration Act and its subsequent amendment in 2004. However, no specific tools were at the time introduced to that end (Facchini et al., 2013). The country's socio-economic problems and the high crime and unemployment rates were attributed to low-skilled African migrants. As a result, barriers to low-skilled migration were entrenched around perceptions of risk and burden (Mbiyozo, 2018). Consequently, restrictive migration policies have featured alongside efforts to manage migration through expanding documentation (Amit and Kriga, 2014). But, as highlighted by Amit and Kriga (2014), the DHA has continually undermined documentation as part of migration management strategy, which has impacted on skilled migrants as well.

Van Lennep (2019) outlines key issues emerging from the Department of Home Affairs (2017) White Paper for International Migration. First, it acknowledges the challenge around attracting and retaining skilled migrants and therefore seeks to ensure skilled migration. Secondly, it prominently features the securitization of migration, which is encapsulated in the need to safeguard “sovereignty, public safety and national security” and a risk-based approach (Department of Home Affairs, 2017). This is evidenced through stricter visa rules for travelers from the African continent and stricter border controls (Abebe, 2019). Thirdly, it establishes, at least outwardly, a pro-African stance. Fourthly, at the expense of newcomers' rights, it enforces control, temporality, and deterrence and, lastly, it expands protectionist measures mainly centered on the integration of migrants.

Though a major destination for migrant laborers entering from the greater African region and beyond, South Africa continues to be characterized by contrasts and contradictions in governmentalities, translating into ambivalent and shifting policies and practices. However, official rhetoric has not always translated into practice. This applies to skilled migration where negative views of migration for skills development continue to persist despite a chronic skills shortage. Policies are key to creating opportunities for accompanying spouses, and this also pertains to LMI (Confurius et al., 2019).

### 2.2. Governmentality and labor market integration

LMI confers numerous benefits for the migrant that are particularly important for females, including economic self-sufficiency and improved socio-economic status (Korteweg, 2017). It can offer a means for accompanying spouses to become less reliant on their spouses, which is especially important in the context of domestic violence (Hiralal, 2017). LMI is also a source of social integration, which shapes the individual's perception of herself as a resource for social identity (Røysum, 2020). LMI can essentially create spaces in which female migrants can be empowered and through which they can achieve modes of being. Research shows that unsuccessful LMI can engender feelings of isolation, alienation, disadvantage or even ignorance of female migrants (Confurius et al., 2019). Critically, LMI is an important source of integration as it is the gateway to other imperative domains of integration such as health, housing, and education. Power relations, however, play an important role around shaping the integration of migrants into the labor market.

This research consulted the governmentality theory to explore the association between the microphysics and the macrophysics of power that influence the labor market outcomes of accompanying spouses within the South African context. Importantly, this research recognizes migrant women as protagonists of their own experiences in tackling the prevalent migration notions that affect them and appropriating them in a manner that is fair and advantageous to their own lives. The governmentality theory has been adapted to different settings, including informal settlements (Massey, 2014), headscarf matters (Teo, 2019), breastfeeding (Malatzky, 2017), and language training policies (Haque, 2017). Within the LMI setting, labor market experiences are the result of conduct of conduct as defined by the theory of governmentality, which produces various subjectivities, particularly during a time when anti-foreign sentiment continues to rise in South Africa (Tshikalange, 2022; Zulu, 2022). This study focused on the conduct of conduct of female accompanying spouses in the Free State, South Africa.

### 2.3. The South African labor market and labor market integration

Labor market assimilation by skilled migrants is based on various preconditions, including labor market conditions and the legislative regulations controlling the labor market (Föbker, 2019). Labor market circumstances differ among nations and regions and can be broadly categorized into two specific groups: (i) adaptable (uncoordinated) and (ii) rigid or inflexible (coordinated). Inflexible markets, such as those found in countries like France, Germany, and the Netherlands, are characterized by strict employment protection legislation (Grigoleit-Richter, 2017; Confurius et al., 2019). In these labor markets, migrants tend to be over-represented as unemployed outsiders due to employers' reluctance to hire unskilled workers (Ballarino and Panichella, 2017; Kesler and Safi, 2018). Kesler and Safi (2018) found that despite the more flexible labor markets in the United Kingdom (UK), Spain and Italy, there tend to be higher earning gaps in these countries due to inequality among migrants employed in high and low service jobs.

The labor market in South Africa can be characterized as rigid (Beukes et al., 2016), considering the stringent employment legislation that governs it. Pertaining to the hiring of immigrants, the Immigration Act (13 of 2002), Section 38(1) stipulates that “no person shall employ (b) a foreigner whose status does not authorize them to be employed by such person, nor (c) a foreigner on terms, conditions or in a capacity different from those contemplated in such foreigner's status” (Department of Home Affairs, 2002). Factors which inhibit employers from hiring immigrants without valid permits or visas include the consequence of fines or possible imprisonment. Authors such as Chinyakata et al. (2019) highlight that the Immigration Act (No. 13 of 2002) contributes to the discrimination faced by immigrants. Strict requirements for the application for a general work permit must include evidence of employment and other documents that justify the selection of migrants over a South African citizen (Department of Home Affairs, 2016).

The primary barrier to LMI for accompanying spouses to South Africa is linked to their visa status. Skilled migrants entering South Africa for work-related purposes are eligible for several visa types, including intracompany visas, permanent residence permits or visas, visas for those in possession of critical skills, and visas for those categorized as general workers who are not considered critical skilled labor. However, their accompanying spouses are allocated either a spousal or dependent visa which does not allow them to work in South Africa (Department of Home Affairs, 2002), thus hindering them from LMI in the host country. The critical skills list, which was adopted in the 2014 Immigration Regulations, outlines the professions that qualify one to obtain a critical skills visa. However, most of the skills regarded as critical and essential are considered male-oriented (Mbiyozo, 2018).

Migrants with foreign skills must submit their qualifications for evaluation by the South Africa Qualifications Authority (2017) so that equivalence can be made against South African standards. Many female migrants, however, are unable to transfer their skills to South Africa (Mbiyozo, 2018). Deemed unskilled, these migrants' ability to integrate into the job market is severely curtailed, and many resort to employment that is below their education or skills levels. Depending on one's occupational niche, the SAQA evaluation may need to be followed up with registration with the relevant professional body like the Engineering Council of South Africa (ECSA) or the Health Professions Council of South Africa (HPCSA), among others (Wojczewski et al., 2015).

On the other hand, South Africa's labor market is characterized by a chronic shortage of skills that has been attributed to poor job growth and economic development. The shortage of skilled workers in South Africa is attributed to a significant brain drain of skilled nationals to countries such as Australia, the UK, Canada, New Zealand, and Germany (Mateus et al., 2014; Phan et al., 2015; Grigoleit-Richter, 2017). The country also faces a huge unemployment challenge, largely attributed to increasing labor market participation but limited generation of employment opportunities, which has seen the unemployment rate soar (Vermaak and Muller, 2019).

South Africa has a gendered labor market that restricts women to certain occupations (Grigoleit-Richter, 2017; Agatiello and Humer, 2018). Statistics South Africa (2018) shows how there is a strong representation of migrant women working in the domestic sector in South Africa, where one out of four female immigrants are employed as domestic workers, even though many of them could be regarded as well educated. Overall, research regarding labor market participation implies better prospects of employment for immigrants than locals, but this not necessarily entails full labor integration (Korteweg, 2017; Vermaak and Muller, 2019), as immigrants are less likely to be employed in work categorized as decent (Statistics South Africa, 2018). The OECD/ILO (2018a) notes growing trends of overqualification both among natives and migrants, and this suggests potential challenges around underemployment among both groups.

Negative attitudes and the racialization of African migrants and othering based on ethnicity or nation of origin are commonplace in South Africa (Adjai and Lazaridis, 2013). Mbiyozo (2018) stresses that the most marginalized and vulnerable African woman migrants in South Africa typically face a triple penalty because of racism, misogyny, and xenophobia. They are often confronted with social exclusion, open hostility, violence, and socioeconomic exploitation [South African Institute of International Affairs (SAIIA), 2008; Hiralal, 2017; Mbiyozo, 2018; Chinyakata et al., 2019], thus imposing barriers to LMI.

## 3. Methodology

### 3.1. Research approach and philosophy

The study used a qualitative approach to obtain profound perspectives of the reality and understanding of the world from the standpoint of the participants. This approach was considered as pertinent to understanding firsthand experiences of the participants from an intersectional perspective. The qualitative case study research design used in this research intended to address the subsequent research question: What are the macro governing policies and factors influencing accompanying spouses in the Free State provinces' integration into the South African labor market? This research was conducted in the South African labor market setting, zoning in on the perspectives of accompanying spouses who were based in the Free State province during the period when the study was conducted. The participants were female accompanying spouses who sought LMI in South Africa at the time. These participants originated from other African nations and migrated to South Africa through family (with their male spouses as lead migrants).

Our ontological stance was to scrutinize the dispositions and opinions of participants that expressed perspectives about their reality. We considered these viewpoints from the interviews that were conducted with the participants (Saunders et al., 2016). We explored and explained the understanding of what is interpreted as the nature of reality by the participants in terms of their everyday lives (Ngulube, 2020). Our epistemological stance was to recognize the place that narratives and individual perceptions hold in the phenomenon of coping with life as an accompanying spouse.

An interpretivist research paradigm was followed in generating knowledge, by uncovering meanings associated with the social phenomenon being explored (Saunders et al., 2016). Knowledge concerning the research phenomenon was founded on the interaction between us and the participants, leading to the emergence of assumptions and themes.

### 3.2. Research population and sampling

Thirteen (13) interviews were conducted in this research. This adhered to the Braun and Clarke's (2021b) suggestions regarding a sufficient qualitative sample size. This study further adopted these scholars' disposition that qualitative studies should place a greater emphasis on the profoundness of the insights gathered rather than on the numbers incorporated in the data collection and analysis processes, as is typically the case with quantitative studies. Thus, we strove to ensure a substantive breadth and depth in terms of the richness of the data collected during the interviews conducted with each participant. It is important to note that while conducting more interviews may have further broadened the scope of the research findings, time and budgetary constraints associated with the fulfillment of the requirements of a graduate qualification prevented this prospect. Additionally, because data was collected during a period when the COVID-19 pandemic still posed a noteworthy threat in the region, a few participants either postponed their appointments, or withdrew completely from the study, which in fact, prolonged the data collection period beyond what had initially been anticipated.

The inclusion criteria adopted in this research were: female accompanying spouses within the age range of 18–65, born outside and not citizens of South Africa, who either initially accompanied or subsequently followed their spouses to the Republic of South Africa to achieve family reunification, who had legal residence status in South Africa, who migrated to South Africa while in possession of a tertiary qualification or work experience, who were currently or formerly employed in South Africa, and who were based in the Free State Province when the study was conducted.

At first, purposive sampling was employed to select participants adhering to the inclusion criteria, through our network of available participants. A complementary snowball sampling approach was then employed whereby participants were requested to refer us to others who met the inclusion criteria of the study. Although not by design, a noteworthy number of the research participants were Zimbabwean citizens (8). We believe this was influenced by the complementary snowball sampling approach, whereby two of the four initially identified participants were Zimbabweans who then mostly referred other Zimbabweans to us. This alludes to the significance of the Zimbabwe-South Africa migration corridor (Crush et al., 2017). The rest of the participants were citizens from various countries in sub-Saharan Africa namely Cameroon, Lesotho, Libya, Nigeria, and Swaziland (Eswatini). In efforts to contribute to the understudied migration research from a South-to-South perspective (Souza and Flippen, 2020), we intentionally excluded female migrants from wealthy countries who have been found to fare much better in terms of achieving LMI in South Africa, than migrants from sub-Saharan Africa (Department of Home Affairs, 2016; Ncube and Mkwananzi, 2020). In Zinatsa and Saurombe (2022a), we also found that European migrants were preferable than other African migrants in South Africa.

At the time of the interviews, the participants were aged between 35 and 52, and all were legally resident in South Africa. Table 1 below outlines other characteristics of the research sample, including the age of the participants upon entry into South Africa, the year they migrated, the highest qualification they possessed when they entered the host country, their highest qualification at the time of data collection, their occupation, and the number of years they each took to achieve labor market integration.

**Table 1:** Characteristics of the research sample.

**Pseudonym**	**Age at entry**	**Highest qualification on entry to South Africa**	**Highest Level of Education currently**	**Field/occupation**	**Year of entry into South Africa**	**Years taken to labor market integration**
Andrea	24	BSc Library Science	Master's in Development Studies	Programme Manager	2007	5
Monica	40	BA (hons) Education Management	PhD in Education Management	Education	2009	2
Iris	29	BSc Animal Science	PhD in Animal Science	Lecturer	2009	3
Priscilla	39	Higher National Diploma in Accounting	PhD in Leadership	Pastor/Businesswoman	2006	9
Palesa	36	BSc Urban and Regional Planning	Master's in Urban Planning	Town Planner	2014	3
Tshepiso	22	BSc Chemistry	PhD in Environmental Management	Environmental Manager	2009	7
Grace	33	BSC Hons Economics	Masters Financial Management	Real Estate	2014	6
Theresa-May	26	National Diploma in Accounting	B Accounting	Quality Analyst	2010	8
Lucille	27	Higher National Diploma Accounting	B(Hons) Accounting	Lecturer	2012	4
Charlotte	22	National Diploma in Journalism	Diploma in Journalism	Admin	2008	7
Unarine	25	Bachelors in Human Resource Management	Bachelors in Human Resource Management	Real Estate	2010	4
Phumzile	21	National Certificate in Purchasing, Supplies and Stores Management	BA Biblical Studies	Pastor	2005	9
Nancy	33	BA Education	Masters in Translation	Teaching Assistant	2016	-

### 3.3. Data collection

The study employed a semi-structured interview guide to delve into various themes concerning labor market integration adapted from extant literature. At first, participants were asked open-ended questions to draw out their narratives relating to their move from their home country to South Africa within the family migration context, while highlighting their labor market trajectory. The questions were designed to explore matters pertaining to structural barriers, labor market conditions and traditional gender roles. More specific questions were subsequently used to probe the participants into providing deeper insights on the subject matter. This phase was used to clarify issues arising from the initial narrative and/or to fill any gaps in the narrative we felt were key to answering the questions of the research study.

Thirteen individual interviews, each lasting ~2 h, were carried out in the English language with female migrants who emigrated to South Africa from other sub-Saharan African countries. Data collection took place online *via* Zoom, between August 2020 and February 2021, in accordance with the COVID-19 social-distancing and other protocols that were in place at the time. Each interview was taped on an external device. Field notes were also made throughout each interview.

The nature of the study and the important ethical issues pertaining to consent and voluntary participation were individually explained to everyone that indicated their willingness to participate. Following verbal consent, each participant was provided with a consent letter to sign and return to us before the pre-determined interview date. Considering the substantial time required for the interviewing process, each one was conducted according to the participants' convenience and availability.

### 3.4. Data analysis

We transcribed the audio-taped interviews in a Microsoft Word document. During data collection and transcription, our engagement with the interview content led to the development of initial ideas regarding coding (Braun and Clarke, 2021a). Braun and Clarke's (2021b) six stages of thematic analysis were adopted for the study as follows: familiarization with the data through immersion, transcription of the data, producing initial codes, the reviewing of themes, the subsequent definition and reviewing of themes, and finally, the report compilation. The complete transcriptions were loaded to ATLAS.ti for analysis and the various cases were compared. Through an iterative process, codes were identified and developed both inductively and deductively from the interviews and the literature, to identify any gaps between the study and existing literature (Braun and Clarke, 2013).

Hadi and Closs's (2015) strategies for ensuring the rigor and quality of the data were adopted in this study as follows:

Self-reflection: We clearly stated their role regarding the study to the participants and were cautious not to give way to any bias that may have resulted from subjective personal viewpoints.

Peer-debriefing: We engaged with two different researchers who were not directly linked to the study but had expertise in similar areas, for the sake of promoting reliability and validity, of course, within the parameters of ethical research conduct.

Extensive description: We offered comprehensive descriptions regarding the context of the research, traits, and characteristics of the sample, as well as the data collection and analysis methods employed in this study, thus enhancing the credibility and possible generalizability (though typically minimal) of the findings to various similar research contexts.

Lengthened engagement: We established rapport and earned the trust of the research participants through engaging with them over a considerable period, which allowed follow up insights to be garnered.

It is important to note that we are both migrants; one primarily being a skilled female accompanying spouse who had not achieved LMI at the time when the study was conducted, and the other, a skilled female migrant who independently achieved full LMI a few years before marriage. Consequently, it was important not to exert our personal experiences or biases in a way that would unduly shape the participants' responses during the interviews. On the other hand, we noticed that the research participants were more comfortable with relaying their experiences to us as blatantly as possible, due to the element of us being able to somewhat relate to their experiences and because they knew they could share their stories with impunity and a lack of judgement as we held no position of power over them. Nonetheless, we strove not to interpret the findings through our own subjective lens, hence, we each analyzed the data separately then later merged our analyses in efforts to ensure that the views of the participants were represented in the most accurate and unbiased manner, a method which proved to be quite effective.

### 3.5. Research ethics and authorization

Informed consent, confidentiality and academic integrity were strictly adhered to in this study. Ethical clearance to conduct this research was granted by the General and Human Research Ethics Committee (GHREC) of the University of the Free State. Ethical clearance code: UFS-HSD2020/0123/0506.

## 4. Findings

This research uncovered an intricate assortment of governing policies and factors functioning mainly at the macro levels through policies and practices. These policies and factors were critical in unequivocally and inadvertently influencing the conduct of accompanying spouses with respect to the labor market in South Africa. Overall, these governing policies and factors seemed to mostly have an adverse effect on the labor market paths of accompanying spouses in South Africa.

Macro-level policies and factors were mostly associated with State and State policies, namely, migration legislation, spouse dependence, the reinforcement of traditional gender roles and restrictive employment legislation which forced the participants' deskilling of qualifications, which were all problematic for LMI.

### 4.1. Theme 1: Migration legislation

Accompanying spouses were eager to secure employment after arriving in South Africa. However, despite their legal residence status in the country, accompanying spouses on spousal visas are not permitted to work in South Africa. This was regarded as one of the greatest impediments to LMI, particularly in the early stages of the labor migration trajectory of many accompanying spouses, as suggested by the following participant:

“… then they realize you have the accompanying spouse visa, then they'll [the employer] just reject the application. So yeah, definitely, it limited the number of opportunities during that time.” (Andrea, Master's in Development Studies, program manager, took 5 years to achieve LMI)

Over and above the creation of barriers to employment, the spousal visa was also regarded as restrictive, in that it set up obstacles around accessing institutional cultural capital (education). This was important for most accompanying spouses, whose skills, upon emigration, could not be categorized as critical or in short supply in the labor market. Studying further was regarded as one of the means of upskilling or upgrading oneself, widening one's employment opportunities and keeping oneself occupied. To enroll in institutions of higher education required one to convert to a study permit. However, qualifications from the country of origin were not always rated equivalent to the South African qualifications (see section on deskilling). Critically, the spousal visa was not only a barrier to education but also to starting a business. Thus, the accompanying visa was regarded as highly restrictive for any form of LMI.

### 4.2. Theme 2: Dependence

Self-determination and independence were viewed as critical to the accompanying spouse's well-being. However, the view of many accompanying spouses was that the spousal visa set up an undesirable pattern of dependence on the lead spouse. On an accompanying visa, getting a driver's license and opening a bank account were subject to the husband's permission or authority. Achieving these things was seen as mostly reliant on the husband willingness and benevolence, as suggested by the participant view that follow:

“… Even if you try and open an account, you have to be with him literally. I have my spouse permit which is written in big letters if I can say that accompanied by [name supplied] with passport number.” (Unarine, Bachelor's in HRM, real estate agent, took 4 years to achieve LMI)

“The thing is with an accompanying spouse permit, if you want to do anything that's legal for yourself, you can't do it without his [spouse] consent. For instance, if I want to go and get a driver's license, he has to sign and say that he has authorized. If I want to open a bank account, he has to sign… There's a lot of restrictions that you have. You are not independent basically. Everything, you must go through the husband.” (Theresa-May, B Accounting, quality analyst, took 8 years to achieve LMI)

The acquisition of permanent residence permits appeared to ease the barriers to LMI but did not guarantee full LMI due to, among other reasons, what was perceived to be the non-desirability of hiring non-South Africans. Most accompanying spouses relied on the acquisition of the permanent residence permits first by the lead spouses. Time to acquisition of spousal-based permanent residence was strongly dependent on the lead spouse's success in acquiring permanent residence and visa processing efficiency. In many instances, the conditionality of the permanent residence applications was also regarded as quite an impediment for self-determination, as implied by the subsequent viewpoints:

“…and probably around 2018, I think my husband applied, ‘cause then he was due for getting a PR. So, we thought, let him apply for his PR first, so that once the PR comes out, we [spouse and children] are all good to go…. We thought now we can apply for the PRs, then my husband's ID was also out.” (Priscilla, PhD in Leadership, pastor/businesswoman, took 9 years to achieve LMI)

“There was a condition that I must remain married to him for the next 2 years after acquiring my PR. If I was to divorce it means it was going to fall away. So, you find out that, that's where a restriction comes in. Let us assume, I was in an abusive marriage, it meant for a woman you had to stay, to wait for that PR or otherwise it was going to affect me to go back and restart again.” (Monica, PhD in Education Management, educationist, took 5 years to achieve LMI)

The pattern of dependence reduced accompanying spouse to an appendage to the lead spouse. In addition to family ties, this pattern of dependence became a greater impediment in regard to accompanying spouses' abilities to pursue any particular course independent from their spouses, as indicated by the following interviewee:

“Being an accompanying spouse. I wish I had just come on my own, looked for my things on my own maybe before I came. I should have established myself.” (Monica, PhD in Education Management, educationist, took 5 years to achieve LMI)

### 4.3. Theme 3: Reinforcement of traditional gender roles

Regarding the huge responsibilities of caring for the extended family, traditional gender roles in which the female was expected to stay at home and do the job of nurturing and household chores was unpreferable and undesirable to accompanying spouses and unfavorable for their families. This was mainly because the core motivation for migration was economical.

Migration legislation was however seen to reinforce traditional gender roles in which the lead spouse (male) was regarded as the sole breadwinner. Most accompanying spouses perceived themselves as being forced into the role of housewife due to the severe restrictions imposed by the migration legislation, a role which some were not familiar with, as evidenced by these perspectives:

“You're just accompanying, you're just there to wash the plates basically and do nothing for yourself.” (Theresa-May, B Accounting, quality analyst, took 8 years to achieve LMI)

“You can't do anything…You literally are just a housewife to be honest.” (Unarine, Bachelor's in HRM, real estate agent, took 4 years to achieve LMI)

Despite being highly skilled, through the accordance of the highly restrictive accompanying spouse visa, many accompanying spouses felt relegated to undertaking reproductive, unpaid care work in the home despite their qualifications and work experience. Due to limited finances arising from a single salary and added responsibilities of caring for the extended family, accompanying spouses were not always able to hire a maid to then take on household and childcare duties. Not hiring a maid was regarded as a means of saving as much money as possible due to the fact that a household would rely on one salary. The burden of the caregiving then automatically fell to the accompanying spouses, as implied by the following statement:

“I mean especially like argh, you are at home already, why would we need help? And sometimes when you really look at it when you get someone who will help you one or two days and they go because I was at home and I was stay home mom. Anyway, we need that extra money.” (Charlotte, Diploma in Journalism, administrator, took 7 years to achieve LMI)

### 4.4. Theme 4: Deskilling of qualifications

At an elementary level, deskilling took place due to the accompanying spouse's mode of migration, but also because of emphasis on skilled migration in South Africa. Despite being educated at a tertiary level, most accompanying spouses found themselves in a situation where their qualifications did not fall within the critical skills shortage lists defined by the Department of Labor. As a result, they could not independently apply for work visas which would significantly have expanded their potential to gain meaningful employment. Not being deemed to dispose of critical skills had more than one consequence: it put them in the unfavorable position of being in direct competition with South African nationals who were being prioritized in the South African labor market; and it made the accompanying spouses reliant on the professionalism and objectivity of the employer around selecting the best qualified person for the job, which according to the participants, was not always honored by employers. Accompanying spouses with critical skills were able to bypass the general work permit route and appeared to have greater ease round entering the labor market.

Deskilling of qualifications was also evident at an institutional level through the South Africa Qualifications Authority (2017) which is the main institution responsible for the verification of foreign qualifications. Deskilling due to this situation particularly impacted on the intention to study further (as a tool for increasing employability) where some accompanying spouses had to “redo” certain qualifications. This had the effect of not only putting them back in terms of time but also increased the curriculum vitae (CV) gaps that eventually did put some of them at a significant advantage when ready to seek employment opportunities, as shown in the following response:

“Funny enough ‘cause I thought I was going to go to masters straight away but they told me no, you know these SAQA qualifications, I don't know how the rating goes by, sometimes the rating is also not so relevant to what we have done… So, in my case I thought I was ready to start with masters. I wrote a proposal, but they said I must start with an honors.” (Monica, PhD in Education Management, educationist, took 5 years to achieve LMI)

Preference for South African citizens (including naturalized citizens) among some employers was attributed to the Broad-based Black Economic Policy, as highlighted in the following statement:

“… you just have to abide to their rules because you know for sure if they employ someone that is a BBBEE candidate, they get something from the government which means if they employ you as a foreigner, they are going to forfeit those benefits…” (Lucille, B(Honors) Accounting, lecturer, took 4 years to achieve LMI)

Altogether, the complex assemblage of macro governing policies and factors appeared to have a largely negative impact on the overall LMI of female accompanying spouses. A strong overarching sense of othering appeared to be the dominant rationality behind most of the strategies employed to keep accompanying spouses out of the labor market.

## 5. Discussion

This research sought to explore the macro governing policies and factors that influence the integration of accompanying spouses into the South African labor market. The research employed a qualitative approach to obtain the viewpoints of accompanying spouses in the Free State, who migrated to South Africa to achieve family unification or re-unification.

Global literature shows that accompanying spouses are a notable influence in attracting skilled migrants within the family migration setting (Bastia and Piper, 2019; Föbker, 2019). Immigration policies that focus on economic growth are criticized for failing to acknowledge the complexities of family migration, particularly, the economic development paradigm fails to attend to the rights, protections, and unique subjectivities of female migrants (Bastia and Piper, 2019). Unsurprisingly, as confirmed by other researchers, including Ncube et al. (2019), economic advantage was a strong push factor for migration from countries of origin for migrant families represented in this study. It is notable that almost half of the migrant families in the study emigrated to South Africa in the period 2007–2009, which suggests the significant impacts of the global economic recession on countries of origin.

Rising unemployment in South Africa, accompanied by the belief that migrants “steal jobs” and the alleged readiness of unskilled migrants to work for lower wages have all contributed to the rise of xenophobic attacks against immigrants (Landau, 2011; Parshotam and Ncube, 2017; Chinyakata et al., 2019; Vermaak and Muller, 2019). These attacks are particularly severe against migrants of African and Asian origin (OECD/ILO, 2018a). Anti-immigrant sentiments continue to be pervasive despite evidence that immigrants do not displace native-born workers and, in fact, make a noteworthy contribution to the economy of South Africa [International Organization for Migration (IOM), 2022]. For instance, migrants contributed nine percent to gross domestic product (GDP) in the year 2011 (OECD/ILO, 2018b). However, South Africa faces a chronic unemployment challenge due to an increasing population but limited industrial expansion. The unemployment rate stood at around 27.7% in 2017 (Statistics South Africa, 2019), which has added to the frustration of locals toward migrants, and while unskilled migrants bear the greater brunt of this frustration, it often also affects skilled migrants who are disdained for their various differences to their local counterparts (such as cultural differences, foreign accents, and a lack of knowledge of the local languages), as demonstrated in another study we conducted (Zinatsa and Saurombe, 2022a). At the same time, the country faces a critical skills shortage which the government acknowledges can be substantially alleviated through the attraction of highly skilled international labor (OECD/ILO, 2018b). Migration scholars in the South African context express their concerns regarding the general hostility toward foreigners which may render migration to the country by skilled international labor unappealing, including where family migration is concerned (Chinyakata et al., 2019; Vermaak and Muller, 2019; Ncube and Mkwananzi, 2020).

Governmentality can shape the subjectivities of the ones governed. For example, Holliday et al. (2019) posit that the categorization of migrant women as dependents, be it by design or by default, shapes their rights in the host country and their ability to exercise those rights. This study found that the males were most likely to be the lead spouses and females were most likely to be dependent on them due to their respective qualifications and experience. In addition, by virtue of having the more desirable skills, lead spouses were able to exert more influence over decision making in the family, as reflected in the statement “it is my husband who steers the ship.” While leading was therefore not necessarily an outcome of traditional gender roles *per se*, the evidence does suggest the subtle and persistent influence of gender roles entrenched in patriarchal rationalities, which does not favor the attainment of the global sustainable development goal of gender equality (United Nations, 2017). Accompanying spouses were mostly placed in a role in which they were following, again, revealing the great dependency of female spouses on male spouses (Ncube et al., 2019).

As indicated in the literature, deskilling is common among migrant women globally, and primarily entails the non-recognition of qualifications attained in one's country of origin (O'Neil et al., 2016; Agatiello and Humer, 2018; Røysum, 2020; Purkayastha and Bircan, 2021; Zinatsa and Saurombe, 2022a). The process of distinguishing skilled migrants from unskilled or semi-skilled ones, and the ascription of value regarding the labor structure, are critical elements of governmentality (Allan and McElhinny, 2017; Del Percio, 2018). At the time of their migration, many of the participants were in their 20 and 30s, which was well within the productive working age in which they could potentially have made a significant contribution to economic development in South Africa. Most of the qualifications that the accompanying spouses held, however, were not aligned to the skills regarded as critical or in short supply to the South African labor market, thus limiting LMI prospects.

Governmentality allows us to understand the macrophysics of power and allows us to see the connections between “power and freedom, resistance and government” (Death, 2016, p. 209). Immigration practices, programs and policies form diverse immigrant groups and essentially influence migrants' rights and identities (power and freedom). Gender inequality in the general global labor market is evidenced by better employment prospects for men, and a significant gender pay gap with lower pay for women in the same jobs as men (Espi et al., 2019; Gereke et al., 2020). Mbiyozo (2018) suggests that one of the challenges in this regard is that most of the skills regarded as critical are male-oriented, thus putting female accompanying spouses at a significant disadvantage by restricting their prospects of LMI. The findings of this study confirm this view. As such, gender inequality and inequity are perpetuated in the labor space. Under South Africa's skilled migration policy regime, those with skills falling outside the category of critical/ special skills are automatically demoted to the level of the deskilled. This makes it difficult for accompanying spouses without these skills to independently acquire permits to work in South Africa.

In non-Western countries, it is common for women to have the duty and responsibility to take care of the home and their children, while men take on the role of breadwinners (O'Neil et al., 2016; Ala-mantila and Fleischmann, 2018). As a result of patriarchal ideologies, women are typically taught to put their household needs above their own aspirations (Phan et al., 2015; Föbker, 2019). Like what was found in Ncube et al.'s (2019) study, the macro governing policies emerging from South Africa's migration legislation can be regarded as particularly detrimental for accompanying spouses' LMI. The adjusted section 11(1)(b)(iv) of the Immigration Act of 2002 provides that the spouse of a South African temporary residence visa holder cannot work, study, or conduct business (Department of Home Affairs, 2017), thus inadvertently promoting traditional gender roles. This study found that, due to the extremely restrictive nature of the migration legislation, particularly regarding the spousal visa, accompanying spouses were relegated to unproductive and unpaid care work in the home. As a result, the migration legislation enforces a situation in which accompanying spouses became appendages to their husbands, whereby they were forced to rely on the latter's benevolence (O'Neil et al., 2016).

Research by Maza (2020) suggests that the period soon after migration is fundamental to future assimilation, both economically and socially. This research suggests that the severe restrictions imposed by the migration legislation, especially pertaining to the spousal visa, do not bode well for future LMI of accompanying spouses. The inability of accompanying spouses to further their studies to expand their capabilities for LMI, while on an accompanying spouse's permit, was established to be problematic, further increasing an initial period of career stagnation. Critically, as other research suggests (Banerjee and Phan, 2015), accompanying spouses were not able to reconcile the gaps, irrespective of their later progress.

Overall, the macro governing policies and factors had the effect of restricting and limiting accompanying spouse's options, and this was detrimental to LMI, particularly, full LMI. Resonant with other global studies (Wojczewski et al., 2015; Britell, 2016; O'Neil et al., 2016), the female migrants in this study experienced lengthy breaks to eventual employment. Various barriers to LMI are indicated in extant literature, for instance, in Zinatsa and Saurombe (2022b), we found that the governing technologies that appeared to be the most difficult to subvert were those relating to immobility on account of family ties and those relating to exclusion, particularly ethnic-based exclusion. The findings of Mbiyozo (2018) regarding this were alike.

For the foreseeable future, South Africa is likely to remain a key hub for immigration in the sub-Saharan region, thus harnessing migration to achieve economic growth will remain a top priority (Department of Home Affairs, 2017). Facilitating the full LMI of accompanying spouses is a key consideration in this regard. The crafting and implementing of gender sensitive policies which consider the intricacies of family migration is necessary, especially those pertaining to the assimilation needs of female migrants. Family friendly policies should look into fostering migration experiences which are pleasant for both the lead and the accompanying spouse. It would also be beneficial for the policy framework to consider the unintended effect of ascribing traditional gender responsibilities through the assigning of “dependent” status to female accompanying spouses, which promotes their redomestication.

Freitas et al. (2015) conducted a study on spouses of Belgian sponsors who were in possession of superior educational qualifications and found that their classification as family migrants and state influence plays a noteworthy role in their successful LMI. This was supported by our findings in Zinatsa and Saurombe (2022b) which revealed that the state—sometimes inadvertently—influences and exacerbates labor market restrictions. A lesson can be learnt from Belgium regarding the more efficient encouragement of family reunification of dependent migrants to the lead migrant, whereby family migrants are expected to provide evidence of integration to have their permit renewed (Purkayastha and Bircan, 2021). This implies the nation's somewhat concern for and continuous evaluation of the support systems and policies that are in place to ensure the successful integration of these migrants and is particularly beneficial for skilled spouses. Ryan and Mulholland (2014) found that networking was often crucial for migrant women to access the labor market and build careers, and this view was corroborated by Zinatsa and Saurombe (2022a) who found that social networks were one of the aids to the successful LMI of migrants. Home countries should try to advise tied migrants concerning the context of South Africa's labor market, employment possibilities, and the tertiary qualifications required to address critical skills shortages in the market, before relocating. This would greatly enhance the possibility of making a stark contribution in the host country and personal envisaged economic outcomes.

The qualitative research methodology does not rely on large samples but rather the depth of insights (Braun and Clarke, 2021b). The sample size in this study was relatively small but sufficient for qualitative research of this nature. Since the study was conducted to satisfy the requirements of a postgraduate qualification, this imposed time and budgetary constraints, hence the data collection period could not be stretched to include more participants. As is the case with most qualitative studies, this study's findings were specific to the research setting and thus would not easily be generalizable to alternate contexts. Since snowball sampling had a noteworthy place in the data collection of this research, participant diversity was diminished regarding age, race, nationality and other biographical aspects, as participants mostly referred others of a similar demographic profile to themselves, to participate in the study. This reduced demographically diverse representation among participants, although we strove to ensure overall scientific rigor. While the study participants were elected based on their legal status as accompanying spouses, it became apparent that, within this category, a significant number of sub-categories resided, thus limiting the findings of the study due to reduced consideration of social stratification.

Recommendations for future research include, a vast quantitative survey or mixed methods inquiry including participants both the male and female genders—investigating the differences between male and female tied migrant experiences could contribute to assessing the specific gender inequalities concerning access to the labor market, and the results could possibly inform policy relating to tied migrants; emphasizing on specific industries, like a typically male-dominated industry, and obtaining the viewpoints of how women experience the labor-market in these industries—investigating the gender inequalities that still exist in male dominated professions and industries, specifically in relation to tied migrants and making policy recommendations that could bridge these inequalities in line with the global sustainable development goals; placing more emphasis on tied migrants who could not ultimately assimilate into the labor market—investigating the encumbrances to LMI among tied migrants who have failed to achieve LMI over prolonged periods of time and making pertinent recommendations to alleviate or resolve their impact; and a comparative research approach focused on reciprocal LMI prospects—investigating the specific economic, social, and other benefits to the host country, of aiding the strategic LMI of tied migrants, based on the country's critical workforce needs and using the results to inform future labor migration policy and legislation.

## 6. Conclusion

The findings of this study support the viewpoint that general reception is fundamental in predicating the results of labor migration. South Africa sets up a unique broadly exclusionary macro context given its continuously heightened limiting migration and employment laws and prevalent immigrant unfriendly disposition. Critically, accompanying spouses are reduced to being housewives and add-ons to their spouses. In this manner, their likelihood of assimilating into the labor market is notably compromized. Accompanying spouses continue to be driven by their individual ambitions informed by how they denote success in the labor market context. Hence, it is important to ascertain ways in which tied migrants could also substantially contribute to the economy of South Africa, rather than merely taking up space.

## Data availability statement

The raw data supporting the conclusions of this article will be made available by the authors, without undue reservation.

## Ethics statement

The studies involving human participants were reviewed and approved by the University of the Free State, General Human Research Ethics Committee. The patients/participants provided their written informed consent to participate in this study.

## Author contributions

This paper was extracted from the doctoral thesis of FZ, who primarily conducted the research, while MDS offered overall guidance regarding all aspects of the study as well as the research conceptualization, general editorial inputs, and the ultimate write-up of the research article. All authors contributed to the article and approved the submitted version.
